# Supplementary material for: The Not so Good, the Bad and the Ugly: Differential Bacterial Adhesion and Invasion Mediated by Salmonella PagN Allelic Variants
Source: Microorganisms. 2020 Mar 30;8(4):489. doi: 10.3390/microorganisms8040489 (PMC7232170; doi:10.3390/microorganisms8040489)
Supplement: Supplementary file 1 [file microorganisms-08-00489-s001.zip › microorganisms-743807-SI/Supplementary Tables/Table S1-Wu-Schifferli.pdf]

Table S1. List of primers for this study.

Primer	Sequence (5' to 3')
pagN$_{Tm+Ty}$_F	TCAGTGATAGAGAAAAGTGCATGAAAAACTTTTTCGCAGTCTGCATCATTCCCCT
pagN$_{Tm+Ty}$_R	CTCATCCGCCAAAACAGCCATTAAAAGGCGTAAGTAATGCCGAGCATGAAGTCATTGG
pagN$_{Di}$_F	TCAGTGATAGAGAAAAGTGCATGAAAAACTTTTTCGCAGTCTGCATCGCTTCCCT
pagN$_{Di}$_R	CTCATCCGCCAAAACAGCCATTAAAAGGCGTAGGTAATGCCGATCATGAAGTCATTGG
pagN$_{Tm}$ *_NdeI*	GGCTGCcatatgAAAGAAGGGATCTATATCACCGGGA
pagN$_{Tm}$ *_HindIII*	CGTCGCaagcttAAAGGCGTAAGTAATGCCGAGCATG
*pagN*_Frag1.F	CCCAGTCTCGAGGTCGACGGTATCGATAAGCTTGATATCGTGGGTCTTCACAACCATGGTC
*pagN*_Frag1.R	AGGCAGGTTCTGAAATGAAAGCCTTTTAAACACTATCGGTAAAATAGCTGG
*pagN*_Frag2.F	ACCGATAGTGTTTAAAAGGCTTTCATTTCAGAACCTGCCTTAATATTGGGCTAAAAGAC
*pagN*_Frag2.R	CTGGAGCTCCACCGCGGTGGCGGCCGCTCTAGAACTAGTGGGGCGTTTCTTTTGGCGC
pagN$_{Ty}$_F1R (D49E)<br>*pagN*$_{Tm}$_F1R (D109Q)	AGTTCTAAACGTACTGGAAGCTGGA
pagN$_{Tm}$_F2F (D49E)<br>*pagN*$_{Ty}$_F2R (D109Q)	TCCAGCTTCCAGTACGTTTAGAACT
pagN$_{D109E}$_F1R	TGTGTACTGGTTCACCAAATGCAATAATATCCTGC
pagN$_{D109E}$_F2F	TGCATTTGGTGAACCAGTACACATAAATGTAAAAAAT
